# Parkin-mediated ubiquitination contributes to the constitutive turnover of mitochondrial fission factor (Mff)

**DOI:** 10.1371/journal.pone.0213116

**Published:** 2019-05-21

**Authors:** Laura Lee, Richard Seager, Yasuko Nakamura, Kevin A. Wilkinson, Jeremy M. Henley

**Affiliations:** School of Biochemistry, Centre for Synaptic Plasticity, Biomedical Sciences Building, University of Bristol, Bristol, United Kingdom; University College London, UNITED KINGDOM

## Abstract

The mitochondrial outer membrane protein Mitochondrial Fission Factor (Mff) plays a key role in both physiological and pathological fission. It is well established that at stressed or functionally impaired mitochondria, PINK1 recruits the ubiquitin ligase Parkin which ubiquitinates Mff and other mitochondrial outer membrane proteins to facilitate the removal of defective mitochondria and maintain the integrity of the mitochondrial network. Here we show that, in addition to this clearance pathway, Parkin also ubiquitinates Mff in a PINK1-dependent manner under non-stressed conditions to regulate constitutive Mff turnover. We further show that removing Parkin via shRNA-mediated knockdown does not completely prevent Mff ubiquitination under these conditions, indicating that at least one other ubiquitin ligase contributes to Mff proteostasis. These data suggest that that Parkin plays a role in physiological maintenance of mitochondrial membrane protein composition in unstressed cells through constitutive low-level activation.

## Introduction

Mitochondria are double membrane-bound organelles that generate 90% of cellular ATP [1]. In most cells, mitochondria form extensive and dynamic networks, undergoing continuous cycles of fission and fusion. This creates a highly adaptable and efficient energy transfer system to rapidly deliver ATP to where it is most needed [2]. In addition, fission plays a central role in the sequestration and selective degradation of defective mitochondria by mitophagy [3, 4]. Mitochondrial fission and fusion are both tightly regulated processes that are largely orchestrated by GTPases. Dynamin and dynamin-related protein (Drp) mediate fission [5, 6] whereas fusion of the mitochondrial outer and inner membranes is driven by the GTPases mitofusins (Mfn) 1 and 2 and Opa1, respectively [7].

Dynamin-related protein 1 (Drp1) is a predominantly cytosolic protein with only ~3% bound to mitochondria under basal conditions [8]. Nonetheless, in cells lacking functional Drp1 the equilibrium between fission and fusion is perturbed, leading to highly elongated and interconnected mitochondria, largely localised in perinuclear clusters [9]. During cell stress, rates of fission and fragmentation increase, causing the release of pro-apoptotic cytochrome *c* from mitochondria, a process that can be delayed by mutation or deletion of Drp1 [10].

Because Drp1 lacks the membrane targeting PH-domain present in conventional dynamins, it requires membrane-bound adaptor/receptor proteins to recruit it to the mitochondrial outer membrane (MOM) [11]. Four mitochondrial Drp1 receptors have been identified; Fis1, MiD49, MiD51 and Mff [12]. Of these, Fis1 is dispensable for mammalian mitochondrial fission [13]. The MiD proteins are specific to higher eukaryotes and although they can each recruit Drp1 to mitochondrial fission sites [14, 15] it remains unclear if MiD proteins facilitate fusion or inhibit fission [16]. Mff facilitates the majority of Drp1 recruitment and is the best characterised Drp1 receptor. It is a ~35kDa protein with a single C-terminal transmembrane domain and interacts with Drp1 via its N-terminus [17]. Like Drp1-null cells, Mff-knockout cells have grossly elongated mitochondria under basal conditions, and attenuated fragmentation and apoptosis following stress [18].

Parkin is a ubiquitin ligase that is inactive in the cytosol but is recruited to damaged/depolarised mitochondria where it is activated by the MOM protein PTEN-induced protein kinase 1 (PINK1). PINK1 is basally maintained at very low levels by rapid proteolytic degradation soon after mitochondrial import [19, 20]. However, loss of membrane potential in damaged or defective mitochondria inhibits PINK1-proteolysis, resulting in its accumulation on the outer membrane, where it phosphorylates mitochondrial ubiquitin at Serine 65 and triggers mitophagy via a multi-step process [21, 22].

Briefly, PINK1-phosphorylated ubiquitin (pUb) binds to and alters the conformation of Parkin. This makes Serine 65 within the Ubiquitin-like domain (UbL) of Parkin accessible for PINK1-mediated phosphorylation, which initiates a cascade of subsequent conformational changes exposing the catalytic site of Parkin [22–24]. In a positive-feedback loop, Parkin ubiquitinates mitochondrial proteins, providing further substrates for PINK1-mediated phosphorylation, which then recruit more Parkin [25, 26]. For example, mitophagy induced by the mitochondrial proton gradient uncoupler carbonyl cyanide m-chlorophenyl hydrazine (CCCP) is largely dependent on Parkin-mediated, non-selective ubiquitination of mitochondrial proteins with K48- and K63-linked ubiquitin chains [27, 28]. Mitochondrial depolarisation leads to PINK1 accumulation on the surface of mitochondria that recruits Parkin to indiscriminately tag MOM proteins with K48- linked ubiquitin chains, marking them for excision and proteasomal degradation [27, 29]. The remaining portion of the mitochondrion is then tagged with K63-linked ubiquitin that recruits phagosomal adaptors including p62 [30] resulting in the engulfment of the organelle into an autophagosome prior to lysosomal fusion and degradation [31, 32]. Thus, this elegant quality control mechanism identifies damaged mitochondria and targets proteins for degradation.

Moreover, in cells lacking functional PINK1 and/or Parkin, mitochondria undergo fragmentation due to excessive Drp1-mediated fission [33–35]. However, the roles of Parkin in non-stressed mitochondria have not been extensively investigated. Here we show that, independent of stress-induced mitophagy, Mff is ubiquitinated by Parkin and at least one other E3 ligase under basal conditions. Our data indicate that Parkin-mediated ubiquitination triggers lysosomal degradation of Mff, suggesting a role for Parkin in homeostatic maintenance of Mff levels and mitochondrial integrity.

## Materials and methods

### Molecular biology

**Fig 1:**
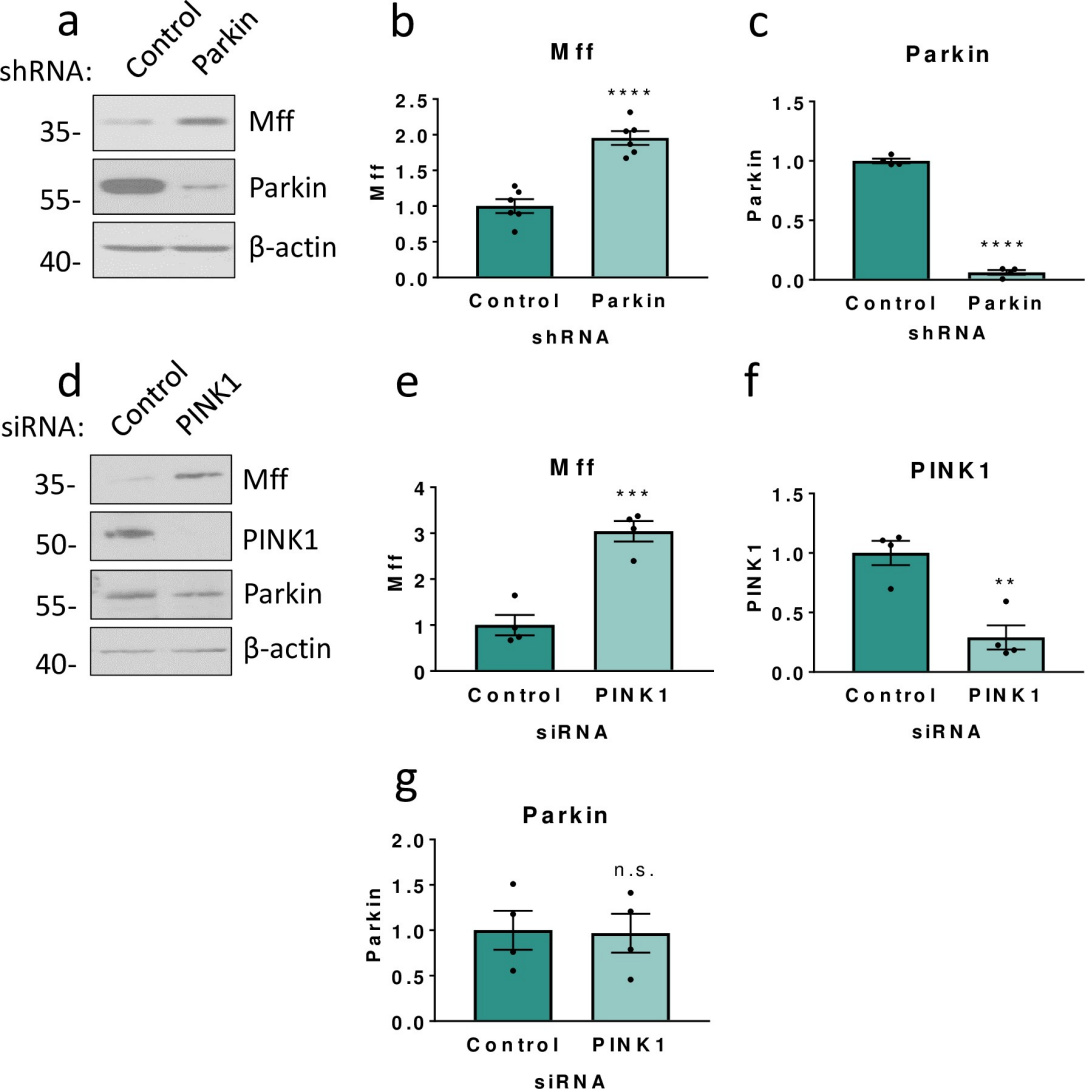
The PINK1/Parkin pathway is involved in Mff stability. a) HEK293T cells were transfected with either shRNA targeting human Parkin or a scrambled control shRNA. Western blots for Mff, Parkin and β-actin. N = 4–7. b,c) Quantitative analysis of Mff and Parkin levels using Student’s unpaired t-test. d) HEK293T cells were transfected with either siRNA targeting human PINK1 or a control siRNA (firefly luciferase). Western blots for Mff, PINK1, Parkin and β-actin. N = 4. e,f,g) Quantitative analysis of Mff, PINK1 and Parkin levels. Data presented as mean ± SEM. ** p < 0.01, *** p < 0.001, **** p < 0.0001.

**Fig 2:**
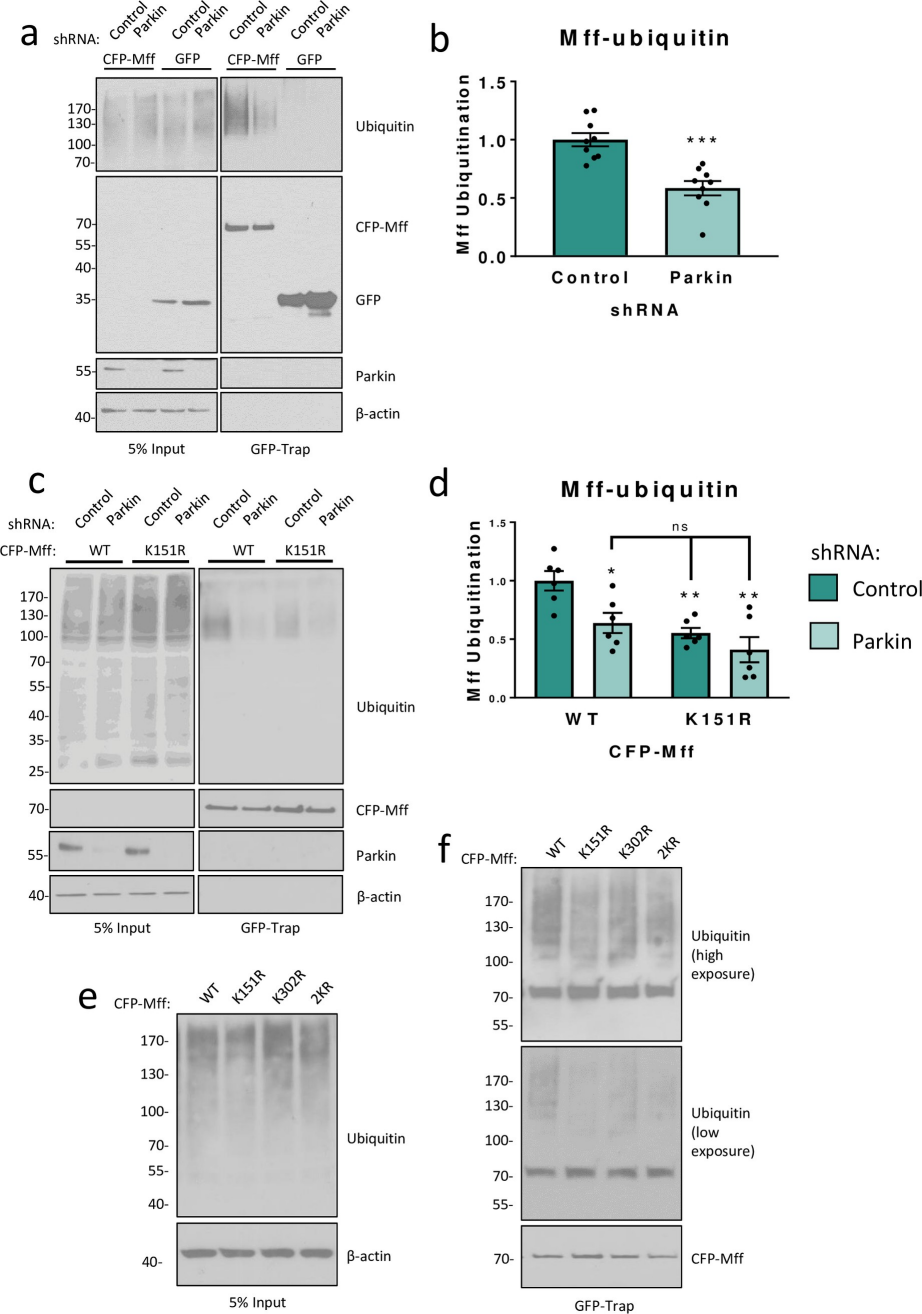
Parkin ubiquitinates Mff at K151 under basal conditions. a) HEK293T cells were co-transfected with CFP-Mff or GFP and shRNA (scrambled control or Parkin-targeting). Western blots of GFP-immunoprecipitation. CFP-tagged Mff immunoprecipitates with endogenous, covalently attached ubiquitin. b) Knockdown of Parkin significantly reduces Mff ubiquitination. N = 9. Analysed using unpaired two-tailed Students’ t-test. Data presented as mean ± SEM. p < 0.001. c) GFP-immunoprecipitations of exogenously expressed CFP-Mff WT or K151R in HEK293T cells reveal that the K151R mutant has significantly reduced endogenous ubiquitination compared to the WT. d) Quantitative analysis showing that knockdown of Parkin significantly reduces ubiquitination of WT CFP-Mff, but not CFP-Mff K151R. N = 6. Analysed using ordinary two-way ANOVA with Tukey’s correction for multiple comparisons with a pooled variance. * p < 0.05, ** p ≤ 0.01. e,f) Input and GFP-immunoprecipitations from HEK293T cells transfected with CFP-Mff WT or mutants showing that K151 and K302 are not the only sites of Mff ubiquitination. Replacement of lysine 151 or 302 with arginine reduces, but does not abolish, ubiquitination. Double replacement of K151 and K302 (2KR) also does not abolish ubiquitination, indicative of at least 1 other site.

**Fig 3:**
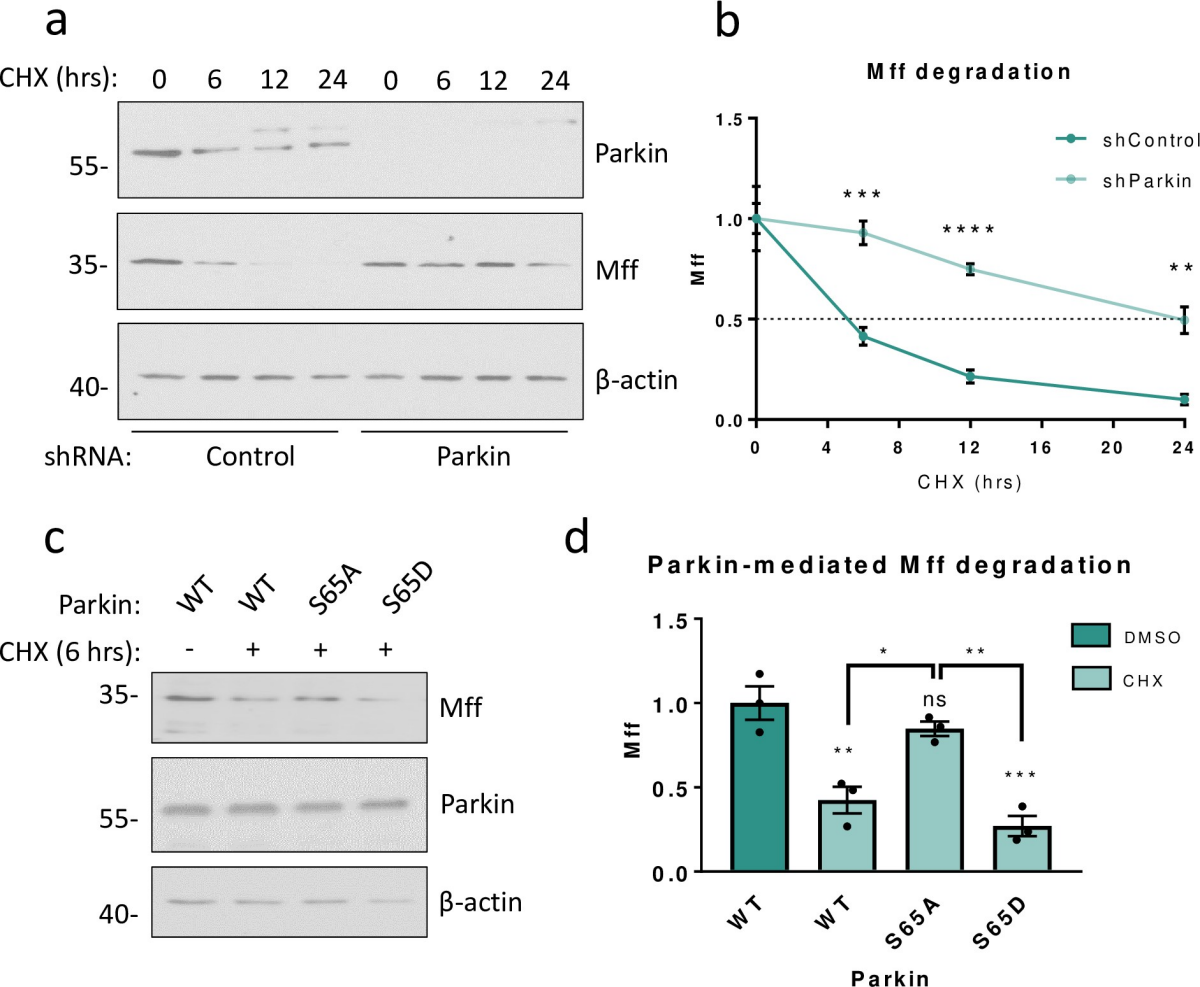
Parkin mediates PINK1-dependent degradation of Mff. a) HEK293T cells were transfected with either scrambled shRNA (control) or shRNA targeting human Parkin. Prior to lysis 72 hours post-transfection, cells were treated with 25μg/mL cycloheximide (CHX) for 0, 6, 12 or 24 hours (0-hour CHX received 24-hour DMSO treatment). Lysates were then Western blotted for Parkin, Mff and β-actin. b) Quantitative analysis of (a), data presented as mean ± SEM. Analysed using unpaired two-tailed Student’s t-tests. N = 4. ** p < 0.01, *** p < 0.001, **** p < 0.0001. c) HEK293T cells were transfected with WT or mutant Parkin. Prior to lysis 48 hours post- transfection, cells were treated with 25μg/mL cycloheximide (CHX) for 6 hours (control treated with DMSO for 6 hours). Lysates were then Western blotted for Mff, Parkin and β-actin. d) Quantitative analysis of (c), data presented as mean ± SEM. Analysed using one-way ANOVA with Tukey’s correction for multiple comparisons with a pooled variance. N = 3. * p < 0.05, ** p < 0.01, *** p < 0.001.

**Fig 4:**
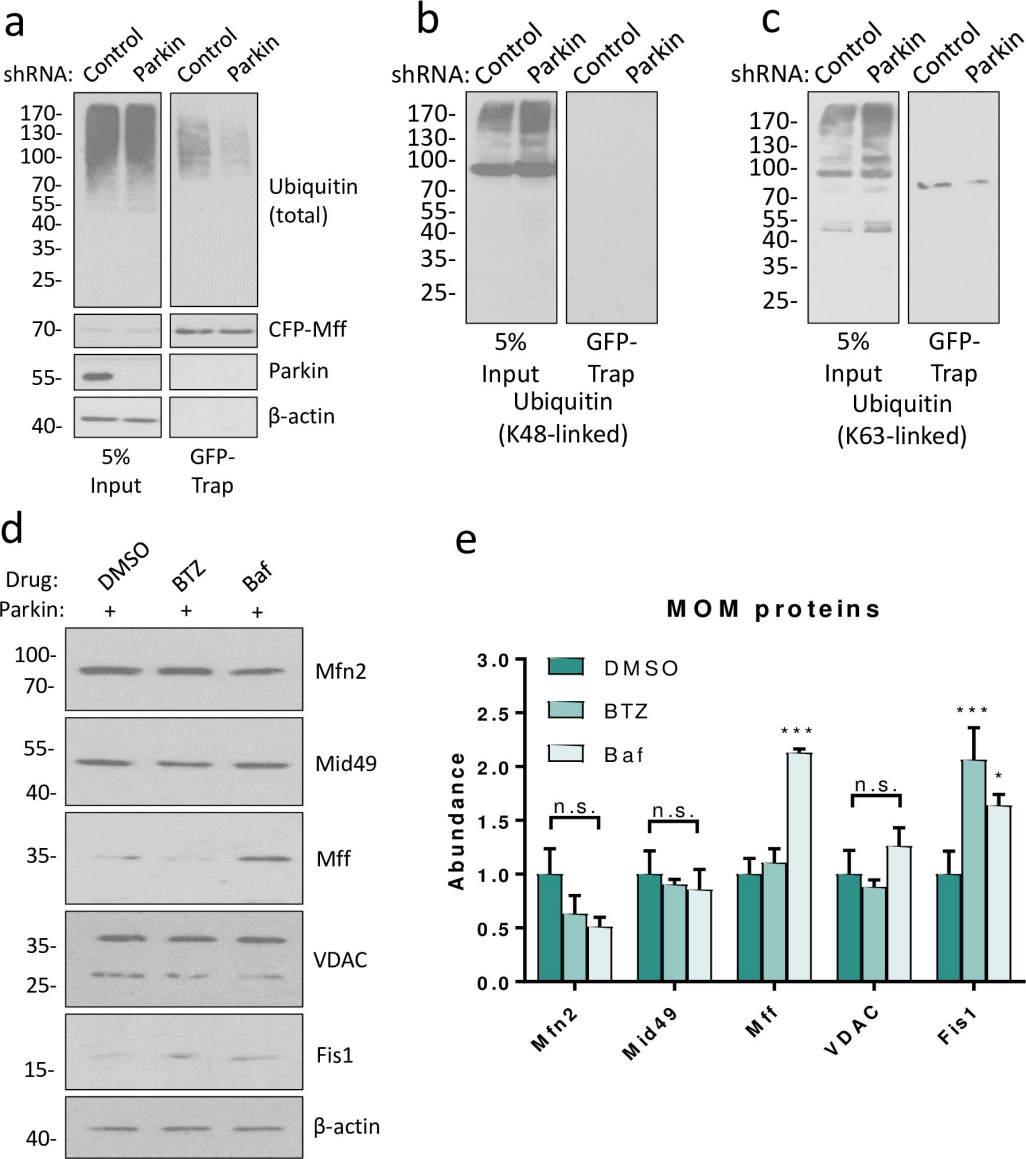
Parkin mediated degradation of Mff is lysosome-dependent. a) HEK293T cells were co-transfected with CFP-Mff or GFP and shRNA (scrambled control or Parkin-targeting). Western blots of GFP-immunoprecipitation. As in Fig 2, knockdown of Parkin reduces total Mff ubiquitination. b) Samples from (a), showing no co-immunoprecipitation of K48-linked ubiquitin with CFP-Mff. c) Samples from (a), showing co-immunoprecipitation of a single K63-linked ubiquitinated CFP-Mff species. d) HEK293T cells were transfected with untagged Parkin. Prior to lysis 48 hours post- transfection, cells were treated with DMSO (control), bortezomib (BTZ, proteasomal inhibitor, 1μM) or bafilomycin (Baf, lysosomal inhibitor, 100nM) for 6 hours. Lysates were then Western blotted for Mff and other mitochondrial membrane proteins. e) Quantitative analysis of (d), data presented as mean ± SEM. Analysed using ordinary two-way ANOVA with Dunnett’s correction for multiple comparisons. N = 3. * p < 0.05, *** p < 0.001.

21bp short hairpin (shRNA) constructs used in Figs 1–4: targeting human shParkin: 5’- ACCAGCATCTTCCAGCTCAAG-3’

non-specific shControl: 5’- AACGTACGCGGAATACTTCGA-3’

were cloned under a H1 promoter into a modified pSUPER vector co-expressing mCherry driven by a PGK promoter. Alternative Parkin shRNAs (S1 Fig) were cloned in the same way, with target sequences:

5’- GCTTAGACTGTTTCCACTTAT-3’ (Parkin (Berger))

5’-AACTCCAGCCATGGTTTCCCA-3’ (Parkin (other)).

Parkin (Berger) target sequence was previously published [36]. Other Parkin shRNA target sequences were designed as part of this study. PINK1 knockdown was performed using MISSION esiRNA human PINK1 (EHU057101, Sigma Aldrich). Mff knockdown (S2 Fig) was performed using siRNA with the target sequence 5’- CCAUUGAAGGAACGUCAGA-3’ (Eurofins genomics). Firefly Luciferase siRNA was used as a negative control (MISSION esiRNA Firefly Luciferase, EHUFLUC, Sigma Aldrich).

The open reading frame of human Mff (isoform I, accession number: Q9GZY8) was cloned into pECFP between 5’ KpnI and 3’ BamHI restriction sites. CFP-Mff expression was driven by a CMV promoter. The open reading frame of human Parkin (full length, accession number: O60260) was cloned into pcDNA3.1(+) between 5’ HindIII and 3’ BamHI restriction sites. Parkin expression was driven by a CMV promoter. CFP-Mff K151R, K302R and 2KR, Parkin S65A and S65D were generated by site-directed mutagenesis.

### HEK293T cell culture and transfection

Human Embryonic Kidney (HEK293T) cells were obtained from The European Collection of Cell Cultures (ECACC). Cultures were maintained at 37°C in a humidified cell culture incubator, supplied with 5% CO2, in Dulbecco’s Modified Eagle’s Medium (Lonza) supplemented with 10% (v/v) Foetal Bovine Serum (Sigma) and 2mM L-Glutamine (Gibco). For transfection, cells were plated on dishes pre-coated with 0.1mg/mL poly-L-lysine to promote adhesion. Lipofectamine 2000 transfection reagent (Invitrogen) was used according to manufacturer’s protocol. Cells were lysed 48 hours (protein over-expression) or 72 hours (protein knockdown) post-transfection.

### Protein biochemistry

For immunoblotting, cells were lysed in sample buffer (1x concentrate) containing 2% SDS (w/v), 5% glycerol (v/v), 62.5mM Tris-HCl pH6.8 and 5% (v/v) 2-β-mercaptoethanol. Lysates were heated to 95°C for 10 minutes prior to gel electrophoresis. For immunoprecipitation, cells were lysed in lysis buffer containing 20mM Tris pH7.4, 137mM NaCl, 2mM sodium pyrophosphate, 2mM EDTA, 1% (v/v) Triton X-100, 0.1% (w/v) SDS, 25mM β-glycerophosphate, 10% glycerol (v/v), 1x cOmplete protease inhibitor cocktail (Roche) and 20mM N-Ethylmaleimide (NEM, Sigma). Lysates were incubated with GFP-Trap agarose beads (ChromoTek) at 4°C for 90 minutes with gentle agitation. Beads were pelleted, washed 3 times with wash buffer (lysis buffer without protease inhibitor cocktail or NEM) and unbound material aspirated. 2x concentrate sample buffer was used to elute immunoprecipitated proteins from the beads. Samples were heated to 95°C for 10 minutes prior to gel electrophoresis.

Denaturing SDS-PAGE was performed on 10–15% (v/v) poly-acrylamide gels. Western blotted PDVF membranes were blocked in 5% (w/v) non-fat milk powder or 4% (w/v) Bovine Serum Albumin (BSA, Sigma) in PBS-T. Primary antibodies used were: Parkin (mouse monoclonal, 1:1000, Santa Cruz sc-32282), Mff (mouse monoclonal, 1:1000, Santa Cruz sc-398731), PINK1 (rabbit monoclonal, 1:1000, Cell Signaling D83G 6946), ubiquitin (mouse monoclonal, 1:1000, P4D1 3936S), Mfn2 (rabbit monoclonal, 1:1000, Cell Signaling, D2D10 9482S), Mid49 (rabbit polyclonal, 1:1000, ProteinTech, I64I3-I-AP), VDAC (rabbit polyclonal, 1:1000, Santa Cruz, FL-283 sc-98708), Fis1 (rabbit monoclonal, 1:1500, ProteinTech, 10956-1-AP), LC3 (rabbit polyclonal, 1:1000, Cell Signaling, 2775S), GFP (rat monoclonal, 1:10,000, Chromotek 3H9), β-actin (mouse monoclonal, 1:10,000, Sigma-Aldrich A5441). For protein detection, membranes were incubated with HRP-conjugated secondary antibodies (1:10,000; Sigma-Aldrich) and visualised by enhanced chemiluminescence. Protein bands were quantified by densitometry using ImageJ (NIH). Mff- and Parkin-antibody specificity was validated by endogenous protein knockdown (S1 and S2 Figs).

## Results

### Knockdown of PINK1 or Parkin increase levels of Mff

We first investigated the effects of Parkin knockdown on Mff by transfecting HEK293T cells with plasmids encoding an shRNA sequence targeted to the human Parkin transcript (shParkin) or a control shRNA sequence. shParkin reduced Parkin levels to 6% of control levels 72 hours after transfection (Fig 1A and 1C). Consistent with Parkin-mediated degradation of Mff there was a corresponding increase in levels of Mff (Fig 1A and 1B). To exclude the possibility of the Mff increase being due off-target effects of the Parkin shRNA, two further Parkin shRNAs were generated and tested in HEK293T cells, with the same effect on Mff levels (S1 Fig).

To determine if the effect of Parkin on Mff levels was PINK1-dependent we next knocked down PINK1 in HEK293T cells [1–3]. 72 hours post-transfection, PINK1 was significantly knocked down to 30% of control values (Fig 1D and 1F). As expected, loss of PINK1 also significantly increased total levels of Mff (Fig 1E). Moreover, total levels of Parkin were unaffected by knockdown of PINK1, indicating that it is Parkin activity, rather than expression, that causes the increase in Mff (Fig 1G).

### Knockdown of Parkin reduces steady-state ubiquitination of Mff

Parkin has been reported to ubiquitinate Mff at K251 in HEK293 cells over-expressing HA-tagged Parkin and treated with CCCP to induce mitochondrial depolarisation [30]. Our data suggest that the PINK1/Parkin pathway may also control Mff levels in the absence of global or applied cell stress. To determine if endogenous Parkin regulates Mff expression under basal, non-stressed conditions, HEK293T cells were co-transfected with CFP-tagged Mff and shRNA targeting Parkin or a scrambled control. 72 hours after transfection, cells were lysed and CFP-Mff retained on GFP-Trap agarose beads. Steady-state levels of Mff ubiquitination were significantly decreased by Parkin knockdown (Figs 2A and 1B). These data demonstrate a role for Parkin in Mff ubiquitination that is not dependent on global mitochondrial stress.

### Parkin ubiquitinates Mff at K151

A recent study has reported that Mff is phosphorylated by AMPK at conserved sites S155 and S172 (highlighted in S2 Fig) [37]. We hypothesised that, given its conservation and density of modifiable residues, this region could be a ‘hotspot’ for Mff post-translational modifications. We therefore selected K151 as an alternative possible target for ubiquitination. To specifically test if Parkin ubiquitinates Mff at K151 under basal conditions, HEK293T cells were co-transfected with CFP-Mff WT or a mutant in which lysine 151 had been replaced with a non-ubiquitinatable arginine (K151R), and either Parkin-targeting or control shRNA. 72 hours post-transfection, cells were lysed and GFP-Trap agarose beads used to precipitate CFP-Mff. Once again, knockdown of Parkin reduced levels of WT Mff ubiquitination (Fig 2C and 2D). However, Parkin knockdown had no significant effect on ubiquitination of CFP-Mff K151R, indicating that under these conditions Parkin preferentially ubiquitinates Mff at K151 (Fig 2C and 2D). Interestingly, while, CFP-Mff K151R had significantly reduced ubiquitination compared to WT, some ubiquitin modification was still present.

The Mff isoform used in the study by Gao et al [30] was isoform II (291 amino acids) which is a truncated protein, lacking a large portion of the N-terminus as well as a central region. Here we used Mff isoform I, which is the full-length protein comprising 342 amino acids. Accounting for these differences in the length of the isoforms, K251 in Isoform II corresponds to K302 in Isoform I (S2 Fig). We therefore generated a CFP-Mff mutant in which lysine 302 was replaced with arginine (K302R). HEK293T cells were co-transfected with CFP-Mff WT or K302R. 48 hours post-transfection, cells were lysed and GFP-Trap agarose beads used to precipitate CFP-Mff. Mutation of Mff K302 to arginine (K302R) reduced, but did not abolish, ubiquitination of Mff isoform I (Fig 2E and 2F). Furthermore, replacing both K151 and K302 with arginines (K151R and K302R; 2KR) also fails to abolish Mff ubiquitination (Fig 2E and 2F). These results indicate that Mff isoform I has several sites of ubiquitination under non-stressed conditions which include K151 and K302, as well as at least one other lysine.

### Parkin mediates PINK1-dependent turnover of Mff

Our results show that Parkin knockdown significantly decreases Mff ubiquitination and increases Mff levels. We next directly tested the role of Parkin in Mff degradation. HEK293T cells were transfected with Parkin-targeting shRNA or a scrambled shRNA control. Prior to lysis 72 hours post-transfection, cells were treated with the protein translation inhibitor cycloheximide (CHX) for up to 24 hours. In cells expressing control shRNA ~90% of Mff was degraded within 24 hours, with a half-life of ~5 hours. In the Parkin knockdown cells, however, the rate of degradation was dramatically slower, increasing the half-life of Mff to ~24 hours (Fig 3A and 3B). Taken together, these data indicate that Parkin-mediated ubiquitination plays a role in physiological Mff degradation under non-stressed conditions.

Parkin ligase activity requires phosphorylation at S65 by PINK1 [22]. To further investigate the roles of Parkin and PINK1 in Mff turnover, we generated phospho-null (S65A) and phospho- mimetic (S65D) untagged Parkin S65 mutants. We reasoned that Parkin S65A would be unable to efficiently translocate to mitochondria or catalyse ubiquitin-transfer due to its PINK1-insensitivity, whereas Parkin S65D would be constitutively active.

HEK293T cells were transfected with WT, S65A or S65D Parkin. Prior to lysis 48 hours post- transfection, cells were treated with CHX or DMSO (vehicle control) for 6 hours. Samples were then Western blotted for Mff (Fig 3C and 3D). Levels of over-expressed Parkin WT, S65A and S65D relative to endogenous Parkin under the same conditions are shown in S3 Fig. Consistent with a PINK1-dependent role for Parkin in Mff turnover, expression of Parkin WT or S65D resulted in significant loss of Mff during 6 hours of inhibited protein translation, whereas Parkin S65A had no effect on Mff levels compared to control. These data demonstrate that PINK1-mediated activation via phosphorylation of Parkin at S65 is required for its activity in Mff turnover.

### Parkin mediates specific lysosomal degradation of Mff

To establish whether constitutive Parkin-mediated degradation of Mff is mediated via the lysosome or the proteasome, we used GFP-Trap immunoprecipitation to pull down CFP-Mff from cells expressing control or Parkin-targeting shRNA. These samples were then probed for total ubiquitin, K48-linked ubiquitin and K63-linked ubiquitin (Fig 4A, 4B and 4C respectively). Indicative of Mff not being a proteasome substrate, no K48-linked ubiquitin was detected on CFP-Mff (Fig 4B). However, a single K63-linked ubiquitin-reactive species was present (Fig 4C), which was reduced in the absence of Parkin, suggesting a role for the lysosome in Parkin-mediated degradation of Mff.

We next investigated the mechanism of Parkin-mediated degradation of Mff by transfecting HEK293T cells with untagged WT Parkin and treating with the proteasomal inhibitor bortezomib [38] or bafilomycin, which inhibits fusion of the autophagosome and lysosome [39] for 6 hours. Over-expression of Parkin WT compared to non-transfected samples is shown in S3B Fig. The efficacy of proteasomal inhibition by 1μM bortezomib was demonstrated by the accumulation of high molecular weight ubiquitin conjugates and a reduction of free ubiquitin compared to DMSO or bafilomycin treatment (S3 Fig). The efficacy of autophagic inhibition by 100nM bafilomycin was confirmed by an increase in LC3-ii/i ratio (S3 Fig). Consistent with Parkin mediating lysosomal degradation of Mff, bafilomycin significantly increased Mff levels, whereas bortezomib treatment had no effect (Fig 4D and 4E). Interestingly, other MOM proteins tested (Mfn2, Mid49, VDAC) were not affected by bafilomycin, while Fis1 was increased by both proteasomal and lysosomal inhibition (Fig 4D and 4E). VDAC and Mfn2 have both been reported to be ubiquitinated by Parkin during induced mitophagy, yet neither were increased by inhibition of mitophagy under the basal conditions of this experiment [32, 40]. These data therefore suggest that under conditions of basal mitophagy, the Parkin-mediated turnover of Mff far exceeds that of other MOM proteins, which could be indicative of an additional selective Parkin-mediated degradative pathway.

## Discussion

Our data show that under basal conditions endogenous Parkin ubiquitinates Mff at K151. For this ubiquitination and subsequent Mff degradation Parkin needs to be activated by PINK1-dependent phosphorylation at S65. This Parkin-mediated ubiquitination of Mff coincides with Parkin-mediated Mff degradation, suggesting that Mff turnover is regulated by a Parkin-dependent, ubiquitin-mediated pathway. Mff is not a substrate of K48-linked ubiquitination but is a substrate of K63-linked ubiquitination. Furthermore, inhibition of the lysosome, but not the proteasome, rescues Mff from Parkin-mediated degradation. These data support a model in which Parkin-mediated degradation of Mff occurs via K63-linked ubiquitination and the lysosome. Interestingly, this activity appears to be in addition to mitophagy, in which depolarised mitochondria recruit Parkin to indiscriminately ubiquitinate MOM proteins prior to their degradation. Of the five MOM proteins assayed in Parkin-overexpressing cells, only Mff and, to a lesser extent, Fis1 were significantly rescued from degradation by inhibition of the lysosome (Fis1 was also rescued by proteasomal inhibition). Mfn2, Mid49 and VDAC were not significantly changed, despite Mfn2 and VDAC being known targets of CCCP-induced, Parkin-mediated mitophagy [32, 40, 41]. These data may indicate that mitophagy is not solely responsible for the changes we observe. Thus, we propose that Parkin may have a selective effect on the turnover of Mff, in addition to its role in mitophagy-dependent Mff degradation. The degradation of Mff by Parkin under basal conditions, together with its inactivity toward other known substrates under the same conditions, suggest that Parkin-mediated degradation of Mff is a regulatory mechanism independent of stress-dependent mechanisms. Moreover, since PINK1 is maintained at low levels in the MOM of healthy mitochondria, we propose that this mechanism plays a critical background role in maintaining mitochondrial integrity in the absence of induced stress.

Intriguingly, our data demonstrate that K302 is not the sole site of ubiquitination of Mff isoform I, and we identified an additional ubiquitination site at K151. However, even in mutants in which both K151 and K302 were ablated, residual Mff ubiquitination remained, indicating the presence of at least one other ubiquitination site. Moreover, our observation that ubiquitination of CFP-Mff K151R is unaffected by Parkin knockdown strongly suggests that the sole, or at least predominant, site of Parkin-mediated ubiquitination under non-stressed conditions is K151.

Our data, combined with previous reports of Mff ubiquitination and phosphorylation [30, 37] indicate Mff is subject to multiple post-translational modifications and suggest that the region around K151 could be an important regulatory ‘hotspot’. Given its role as the primary receptor for Drp1 in mitochondrial fission, further work will be needed to elucidate how these modifications, and the interplay between them, regulate Mff abundance and activity in health and disease.

## Supporting information

S1 Fig Validation of Parkin antibody and knockdown constructs. a) Parkin antibody and shRNA are specific. HEK293T cells were transfected with control (scrambled sequence) shRNA or shRNA targeting human Parkin. Cells were lysed 72 hours post-transfection and lysates used for Western blotting with anti-Parkin antibody (Santa Cruz sc-32282). A single band of around 55kDa was detected (predicted MW: 52kDa), which was abolished by Parkin shRNA.
b) Effect of Parkin knockdown on Mff is specific. HEK293T cells were transfected with control (scrambled sequence) shRNA or one of 3 shRNAs targeting human Parkin. Cells were lysed 72 hours post-transfection and lysates used for Western blotting with anti-Parkin antibody (Santa Cruz sc-32282) and anti-Mff antibody (Santa Cruz sc-398731). shRNA construct target sequences: Parkin (blue) 5’-ACCAGCATCTTCCAGCTCAAG-3’, Parkin-Berger (purple) 5’-GCTTAGACTGTTTCCACTTAT-3’, Parkin-other (red) 5’-AACTCCAGCCATGGTTTCCCA-3’. Parkin (Berger) shRNA target sequence taken from [4].
c) Partial sequence alignments of human Parkin (Uniprot: O60260) and shRNAs, coloured as in a) and b).
(TIF)

S2 Fig Mff isoform alignment. a) Mff antibody is specific. HEK293T cells were transfected with control (Firefly luciferase) siRNA or human Mff siRNA (5’-CCAUUGAAGGAACGUCAGATT-3’, Eurofins genomics). Cells were lysed 72 hours post-transfection and lysates used for Western blotting with anti-Mff antibody (Santa Cruz sc-398731). No bands were detected in Mff knockdown cells.
b) Alignment of all five isoforms of human Mff. Isoform I (green, 342 amino acids) is the longest and was used to generate CFP-Mff constructs. Isoform II (orange, 291 amino acids) was used in the study by Gao et al. Residue numbers are given according to their position in Isoform I. S155 and S172 are present in all isoforms of Mff (red). K151 (blue) and K302 (purple) are also present in all isoforms. Alignment produced using ClustalOmega. Uniprot identifiers are as shown (Q9GZY8).
(TIF)

S3 Fig Parkin over-expression and further controls for Figs 3 and 4. a) Samples from Fig 3C (over-expressing Parkin WT, S65A or S65D, in the presence of CHX) alongside non-transfected HEK293T cells in the presence of CHX. Lysates probed for Parkin, Mff and β-actin.
b) Samples from Fig 4D (over-expressing Parkin WT, in the presence of DMSO, BTZ or Baf) alongside non-transfected HEK293T cells in the presence of DMSO. Lysates probed for Parkin, ubiquitin, LC3 and β-actin.
c) Quantitative analysis of (c), data presented as mean ± SEM. Analysed using ordinary one-way ANOVA with Tukey’s correction for multiple comparisons with a pooled variance. N = 3. * p < 0.05.
(TIF)
