# Supplementary figures and images for: Parkin-mediated ubiquitination contributes to the constitutive turnover of mitochondrial fission factor (Mff)
Source: PLoS One. 2019 May 21;14(5):e0213116. doi: 10.1371/journal.pone.0213116 (PMC6528996; doi:10.1371/journal.pone.0213116)

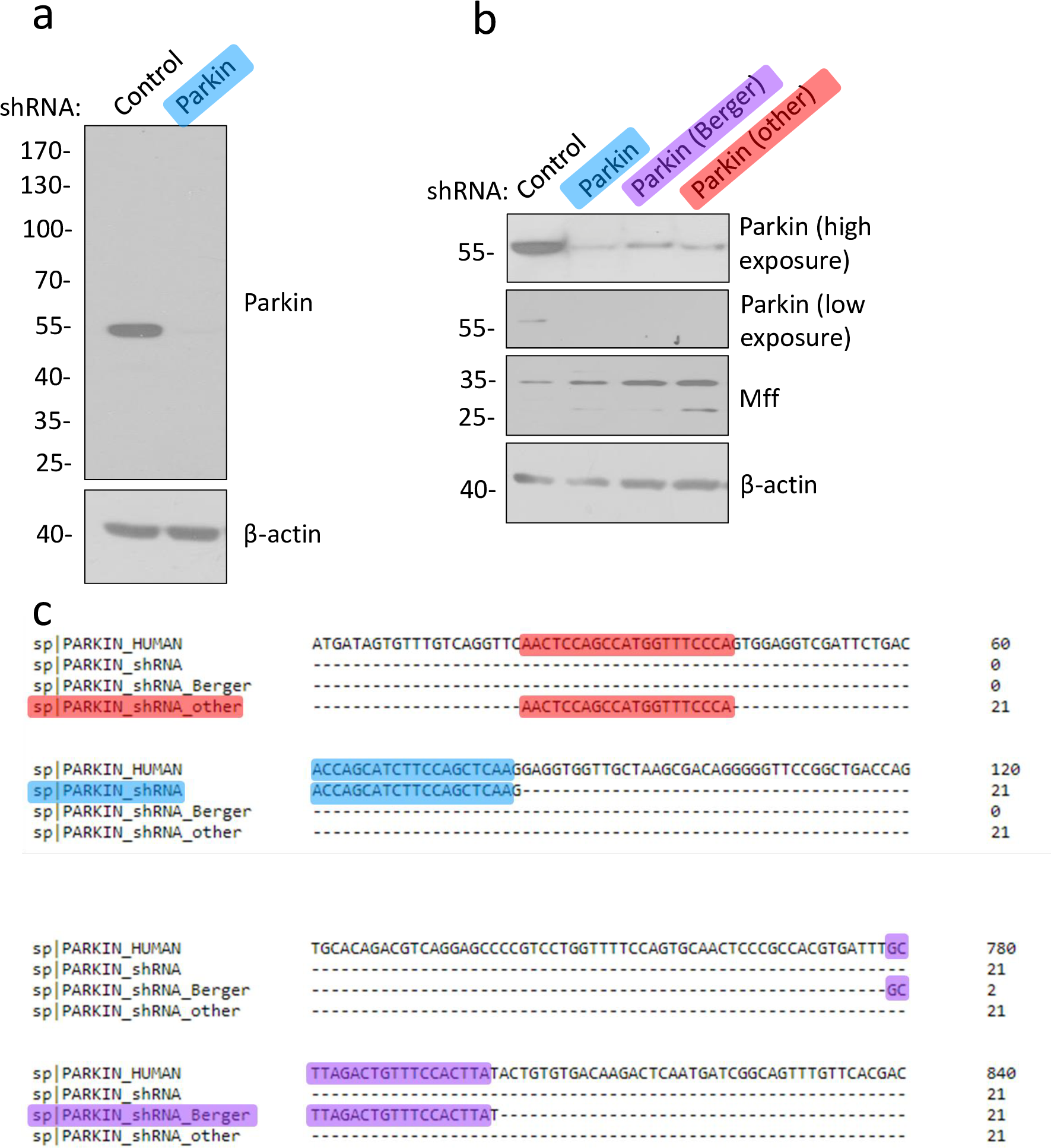

Supplement: S1 Fig — a) Parkin antibody and shRNA are specific. HEK293T cells were transfected with control (scrambled sequence) shRNA or shRNA targeting human Parkin. Cells were lysed 72 hours post-transfection and lysates used for Western blotting with anti-Parkin antibody (Santa Cruz sc-32282). A single band of around 55kDa was detected (predicted MW: 52kDa), which was abolished by Parkin shRNA. b) Effect of Parkin knockdown on Mff is specific. HEK293T cells were transfected with control (scrambled sequence) shRNA or one of 3 shRNAs targeting human Parkin. Cells were lysed 72 hours post-transfection and lysates used for Western blotting with anti-Parkin antibody (Santa Cruz sc-32282) and anti-Mff antibody (Santa Cruz sc-398731). shRNA construct target sequences: Parkin (blue) 5’-ACCAGCATCTTCCAGCTCAAG-3’, Parkin-Berger (purple) 5’-GCTTAGACTGTTTCCACTTAT-3’, Parkin-other (red) 5’-AACTCCAGCCATGGTTTCCCA-3’. Parkin (Berger) shRNA target sequence taken from [4]. c) Partial sequence alignments of human Parkin (Uniprot: O60260) and shRNAs, coloured as in a) and b). (TIF) [file pone.0213116.s001.tif]

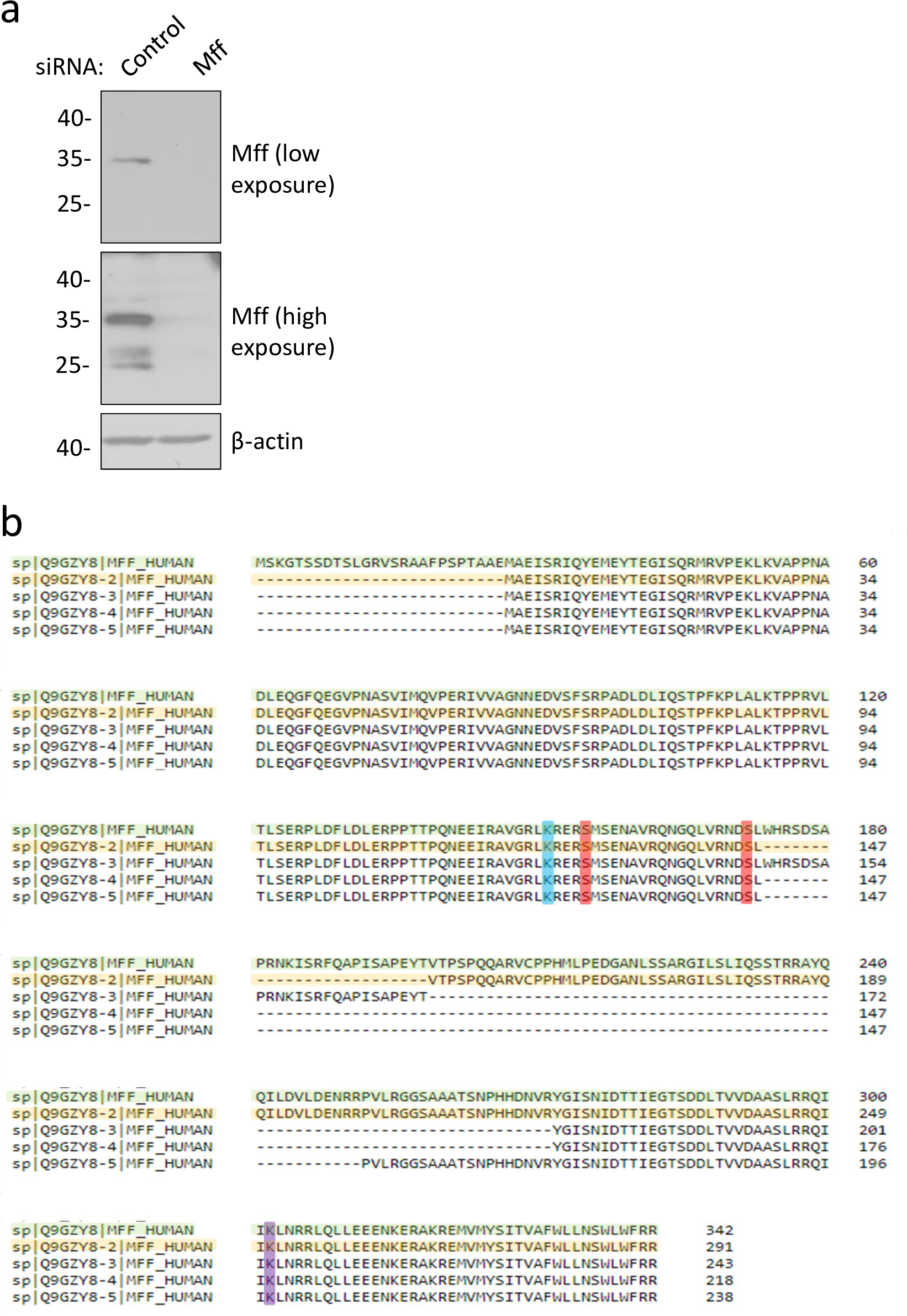

Supplement: S2 Fig — a) Mff antibody is specific. HEK293T cells were transfected with control (Firefly luciferase) siRNA or human Mff siRNA (5’-CCAUUGAAGGAACGUCAGATT-3’, Eurofins genomics). Cells were lysed 72 hours post-transfection and lysates used for Western blotting with anti-Mff antibody (Santa Cruz sc-398731). No bands were detected in Mff knockdown cells. b) Alignment of all five isoforms of human Mff. Isoform I (green, 342 amino acids) is the longest and was used to generate CFP-Mff constructs. Isoform II (orange, 291 amino acids) was used in the study by Gao et al. Residue numbers are given according to their position in Isoform I. S155 and S172 are present in all isoforms of Mff (red). K151 (blue) and K302 (purple) are also present in all isoforms. Alignment produced using ClustalOmega. Uniprot identifiers are as shown (Q9GZY8). (TIF) [file pone.0213116.s002.tif]

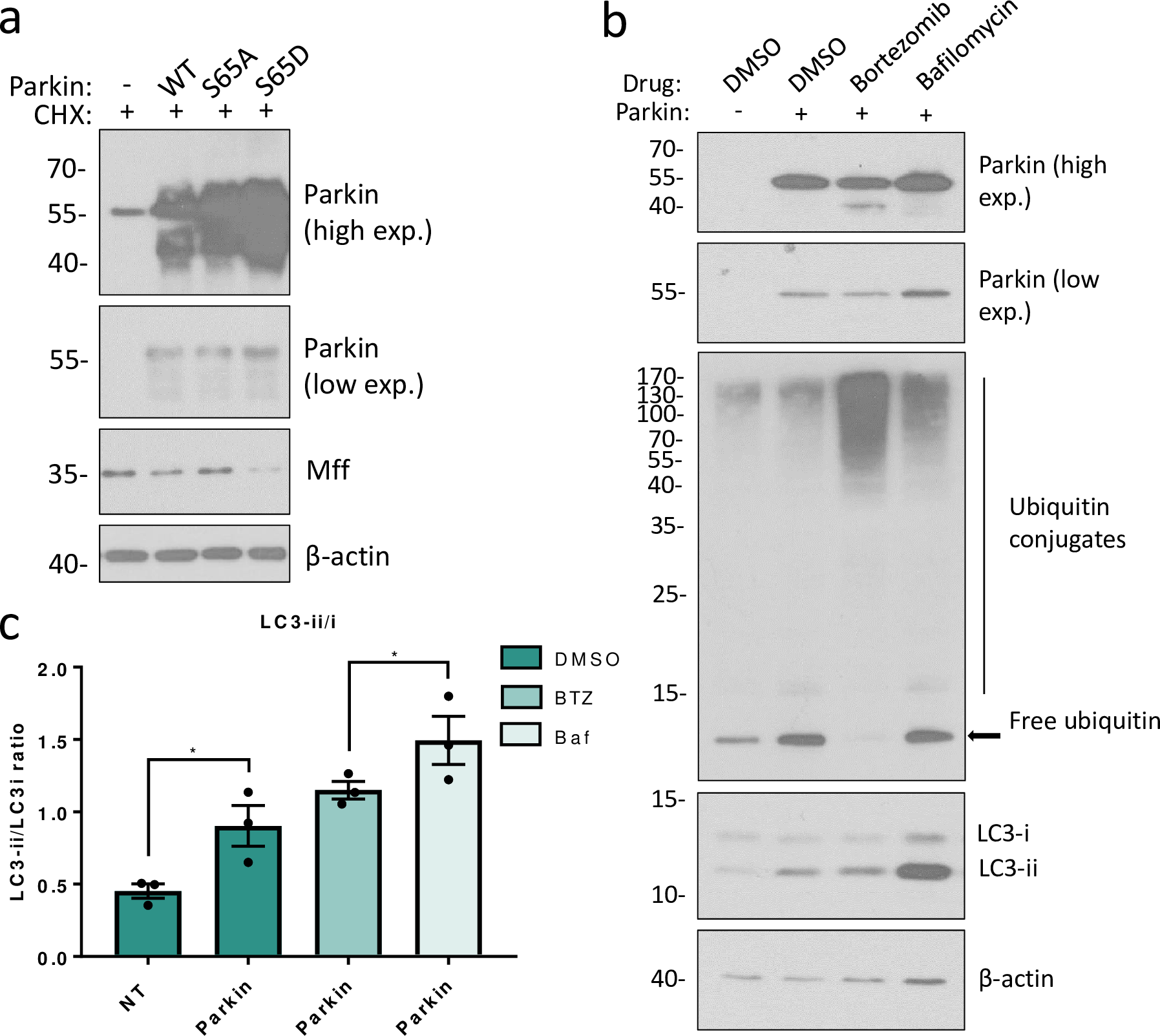

Supplement: S3 Fig — a) Samples from Fig 3C (over-expressing Parkin WT, S65A or S65D, in the presence of CHX) alongside non-transfected HEK293T cells in the presence of CHX. Lysates probed for Parkin, Mff and β-actin. b) Samples from Fig 4D (over-expressing Parkin WT, in the presence of DMSO, BTZ or Baf) alongside non-transfected HEK293T cells in the presence of DMSO. Lysates probed for Parkin, ubiquitin, LC3 and β-actin. c) Quantitative analysis of (c), data presented as mean ± SEM. Analysed using ordinary one-way ANOVA with Tukey’s correction for multiple comparisons with a pooled variance. N = 3. * p < 0.05. (TIF) [file pone.0213116.s003.tif]
